# Characterization of the tumor immune microenvironment in pregnancy-associated breast cancer through multiplex immunohistochemistry and transcriptome analyses

**DOI:** 10.1186/s13058-025-02097-4

**Published:** 2025-08-26

**Authors:** Ching-Hsuan Chen, I-Chun Chen, Chia-Lang Hsu, Tzu-Pin Lu, Ming-Yang Wang, Li-Wei Tsai, Chiun-Sheng Huang, Yen-Shen Lu, Ching-Hung Lin

**Affiliations:** 1 Institute of Epidemiology and Preventive Medicine, College of Public Health, National Taiwan University, Taipei, Taiwan; 2 Department of Obstetrics and Gynecology, Taipei City Hospital Heping Fuyou Branch, Taipei, Taiwan; 3 Department of Medical Oncology, National Taiwan University Cancer Center, Taipei, Taiwan; 4 Graduate Institute of Oncology, National Taiwan University, Taipei, Taiwan; 5 Department of Oncology, National Taiwan University Hospital, Taipei, Taiwan; 6 Department of Medical Research, National Taiwan University Hospital, Taipei, Taiwan; 7 Institute of Health Data Analytics and Statistics, College of Public Health, National Taiwan University, Taipei, Taiwan; 8 Department of Surgical Oncology, National Taiwan University Cancer Center, Taipei, Taiwan; 9 Department of Surgery, National Taiwan University Hospital, Taipei, Taiwan; 10 Department of Internal Medicine, National Taiwan University Hospital, Taipei, Taiwan

**Keywords:** Pregnancy, Breast cancer, Immune microenvironment

## Abstract

**Background:**

Pregnancy-associated breast cancer (PABC) is breast cancer diagnosed during pregnancy or within 2 years postpartum. Although relatively rare, it is associated with a poor prognosis, and the underlying mechanisms contributing to this unfavorable condition remain incompletely understood. In this study, we investigated tumor microenvironmental features linked to pregnancy and lactation in an effort to elucidate these mechanisms.

**Methods:**

This retrospective study included 26 patients with PABC, 51 patients with breast cancer diagnosed 2–5 years postpartum (post-weaning breast cancer [PWBC]), and 28 patients with no prior history of pregnancy at the time of breast cancer diagnosis (nulliparous breast cancer [NPBC]). The tumor immune microenvironment in PABC, PWBC, and NPBC cases was profiled using Opal Polaris 7 color immunohistochemistry (IHC) and the NanoString Breast Cancer 360 Gene Expression Panel.

**Results:**

No significant differences in tumor stage or molecular subtype were observed among the PABC, PWBC, and NPBC groups. The age of diagnosis was comparable between NPBC and PABC patients (38.0 vs. 35.4 years), but significantly higher in the PWBC group (42.2 years). Both multiplex IHC and transcriptomic analyses consistently demonstrated that the PABC and PWBC groups exhibited a higher abundance of tumor-infiltrating immune cells than the NPBC group. Specifically, multiplex IHC analysis revealed that PABC and PWBC were associated with increased densities of CD4^+^, CD8^+^, CD20^+^, and CD68^+^CD163^+^ cells. Consistently, transcriptomic analysis indicated that the PABC and PWBC groups exhibited elevated gene expression signatures associated with macrophages, cytotoxic cells, CD8^+^ T cells, and B cells compared with the NPBC group. The primary differences observed between the PABC and NPBC groups were validated using three publicly available datasets from the Gene Expression Omnibus.

**Conclusions:**

Using multiplex IHC and transcriptome analyses, this study demonstrated that PABC was associated with a higher abundance of immune cells, including increased infiltration of T cells, B cells, and macrophages, in the breast tumor microenvironment. Future research is required to focus on the role of immune cells in pregnancy-associated breast cancer patients.

**Supplementary Information:**

The online version contains supplementary material available at 10.1186/s13058-025-02097-4.

## Background

Pregnancy-associated breast cancer (PABC) is a subset of breast cancer that develops during pregnancy or within a few years postpartum [1]. PABC occurs in approximately 1 in every 5000 deliveries and represents the most prevalent malignancy associated with pregnancy [2, 3]. As the maternal age at childbirth has increased, the incidence of pregnancy-associated cancers, including PABC, has also increased [2, 4]. Evidence indicates that the prognosis for patients with PABC is poorer than that for those with nulliparous breast cancer (NPBC), and this effect may persist in individuals who receive a diagnosis several years after childbirth [5, 6]. A meta-analysis reported a 50% higher mortality hazard in women with a breast cancer diagnosis within 2 years postpartum compared with that in nulliparous women [5]. Furthermore, research reported that the 5-year overall survival rate was significantly lower in patients with PABC than in those who received a breast cancer diagnosis at 5 years after delivery (90% vs. 95%) [6].

The poor prognosis associated with PABC may be attributable to advanced disease stage at diagnosis, a higher prevalence of aggressive tumor subtypes, and delays in initiating treatment; common physiological changes during pregnancy, such as nipple discharge and breast lumps, may obscure the early clinical signs of breast cancer, thereby contributing to delayed diagnosis and more advanced disease presentation. Additionally, PABC has been associated with a higher prevalence of triple-negative breast cancer and a lower prevalence of hormone receptor–positive subtypes [6–8]. Despite these associations with tumor stage, tumor subtype, and other prognostic factors, the extent to which pregnancy itself adversely affects prognosis remains a subject of ongoing debate. Some studies on breast cancer diagnosed during pregnancy have reported no significant difference in survival outcomes after adjustment of tumor stage, tumor grade, and other variables in Cox regression analyses [8, 9]. However, these studies have also reported trends toward increased risk [8, 9], and a population-based study reported a significantly worse adjusted hazard ratio for PABC [6].

The tumor immune microenvironment has been identified as a critical prognostic and predictive factor in cancer therapy. The mammary gland undergoes extensive physiological changes during pregnancy, lactation, and the subsequent phases of postpartum remodeling and involution. Evidence from preclinical animal models has indicated that the remodeling of the mammary gland to its prepregnant state activates wound-healing-like biological programs. This remodeling process is characterized by an initial proinflammatory response followed by an immunosuppressive phase [10].

Advanced methodologies such as multiplex immunohistochemistry (IHC) and transcriptomic profiling provide robust and effective means for characterizing the tumor immune microenvironment, even in archival formalin-fixed paraffin-embedded (FFPE) tumor specimens from rare disease entities. Therefore, the current study employed these techniques to investigate the tumor immune environment in PABC and postpartum breast cancer and identified distinct immunological features in PABC relative to non-PABC.

## Methods

### Patient population

This retrospective study analyzed 105 archival FFPE tumor tissue samples obtained from young women aged < 50 years with primary invasive breast cancer diagnosed at National Taiwan University Hospital between January 2004 and August 2016. Clinicopathological data were extracted from electronic medical records. During the study period, 26 patients were given a diagnosis of breast cancer either during pregnancy or within 2 years postpartum and were classified into a PABC group. An additional 51 patients were given a diagnosis of breast cancer 2–5 years after delivery and were categorized into a PWBC group. The NPBC group comprised 28 age- and tumor stage–matched patients with no history of pregnancy before their breast cancer diagnosis. The study protocol was approved by the Ethics Committee of National Taiwan University Hospital (Approval No. 201711051RINC). Available pathology slides and tumor blocks from the hospital’s pathology department were collected for analysis.

### Multiplex immunohistochemistry analysis

Immune profiling at the protein level was conducted using the Opal 7 Immunology Discovery Kit (OP7DS2001KT, Akoya Biosciences). Sections measuring 4 μm in thickness were obtained from FFPE tumor blocks, deparaffinized with xylene, and subsequently rehydrated with a series of gradient ethanol solutions. Antigen retrieval was performed using an AR6 or AR9 buffer for 15 min using microwave treatment. The Opal multiplex protocol involved six sequential biomarker–Opal fluorophore staining cycles on the same slide with the following antibody–fluorophore pairings: CD4–Opal 520, CD163–Opal 540, CD8–Opal 570, CD20–Opal 620, CD68–Opal 650, and PanCK–Opal 690. Three additional antibodies were selected for use in the open channel: CD163 (clone 10D6, MA5-11458; Thermo Fisher Scientific), CD20 (clone L20, 61-0010-5; Genemed), and PanCK (MA5-13156; Thermo Fisher Scientific). Nuclear counterstaining was performed with 4′,6-diamidino-2-phenylindole (DAPI), followed by mounting with ProLong Diamond Antifade Mountant (P36965; Thermo Fisher Scientific). Slide imaging was performed using the Vectra Polaris Automated Quantitative Pathology Imaging System (Akoya Bioscience, formerly PerkinElmer), and tumor regions of interest were selected using Phenochart Software. Finally, inForm Tissue Analysis Software was employed to separate overlapping multiplexed immunofluorescence signals and analyze immune profiles. The extent of colocalization between immune cells and tumor cells was assessed using the normalized spatial intensity correlation (NSInC) index and the immune cells served as base signals [11]. The NSInC index ranges from − 1 to 1, with 1 indicating perfect colocalization, 0 representing no correlation, and − 1 denoting perfect dispersion.

### Tumor RNA isolation and transcriptomic analysis

RNA was isolated from FFPE tumor tissue using the Roche High Pure FFPE RNA Isolation Kit (Roche Molecular Systems, Pleasanton, CA, USA). To ensure sample purity (optical density at 260/280 nm, ratio range, 1.7–2.5), RNA concentration was measured using a Nanodrop ND-1000 spectrophotometer and a Qubit 3.0 fluorometer (Thermo Fisher Scientific, Waltham, MA, USA). Gene expression profiling was performed using the NanoString Breast Cancer 360 (BC360) Panel (NanoString Technologies, Seattle, WA, USA), which included 776 human genes across 48 gene signatures associated with molecular subtypes and immune cell abundance. Raw transcriptomic data were processed and extracted using the nSolver analysis software (NanoString Technologies) and the ROSALIND Platform. Molecular subtyping of breast cancer was conducted utilizing the prediction analysis of microarray 50 (PAM50) classifier [12]. Immune cell signatures were derived using the nSolver advanced analysis module, which incorporated R version 3.3.2.

### Validation using external datasets

Three publicly available datasets—GSE31192, GSE53031, and GSE112825—were obtained from the Gene Expression Omnibus (GEO) database [13–15] to validate our findings. Because the series matrix file for GSE112825 contained only partial array data, raw CEL files were retrieved and processed using a robust multiarray method [16]. The tumor immune environment was assessed using a 547-gene Cell-Type Identification by Estimating Relative Subsets of Known RNA Transcripts (CIBERSORT) algorithm [17]. The CIBERSORT absolute immune score was calculated as the sum of all imputed immune cell fractions. For comparative analysis, the Opal immune score was defined as the sum of immune cell densities (CD4⁺, CD8⁺, CD20⁺, CD68⁺, CD68⁺CD163⁺) measured by multiplex IHC, while the BC360 immune score represented the aggregated immune cell signals (B cells, CD8 T cells, cytotoxic cells, exhausted CD8 T cells, macrophages, mast cells, neutrophils, and regulatory T cells) derived from transcriptomic profiling.

### Statistical analysis

Overall immune cell distributions were compared using permutational multivariate analysis of variance based on Euclidean distance metrics. Between-group numerical comparisons were performed using the Wilcoxon rank-sum test and the Kruskal‒Wallis test, as appropriate. Categorical variables were analyzed using Fisher’s exact test. Kaplan Meier curve with log rank test was used for survival analysis. Hazard ratios were estimated with Cox regression. To account for multiple comparisons, *p* values were adjusted using the Holm‒Bonferroni method. A *p* value of < 0.05 was considered significant. All statistical analyses were conducted using R version 4.2.1.

## Results

### Characteristics of study population

Table 1 presents a summary of the demographic and clinical characteristics of the study population. The mean age at breast cancer diagnosis was 38.0 years in the NPBC group, 35.0 years in the PABC group, and 42.2 years in the PWBC group (*p* < 0.001). The age difference between groups was primarily attributable to the PWBC group, which was significantly older than both the NPBC (*p* = 0.04) and PABC (*p* < 0.001) groups in pairwise comparisons. However, no significant difference in patient age was observed between the NPBC and the PABC groups (*p* = 0.22). Regarding disease stage, 31 patients (29.5%) had stage I, 49 (46.7%) had stage II, and 35 (33.3%) had stage III breast cancer. Molecular subtyping by PAM50 classification revealed 12 basal-like, 25 HER2-enriched, 26 luminal B, and 42 luminal A tumors. No significant differences in disease stage (*p* = 0.156) or molecular subtype (*p* = 0.636) were observed among the NPBC, PABC, and PWBC groups.

**Table 1:** Patient characteristics

	NPBC *N* = 28	PABC *N* = 26	PWBC *N* = 51	*p* value
Age (Mean ± SD)	38.0 ± 5.3	35.4 ± 6.3	42.2 ± 6.5	< 0.001
Stage --- N (%)				
I II III	6 (21.4) 12 (42.9) 10 (35.7)	5 (19.2) 16 (61.6) 5 (19.2)	20 (39.2) 21 (41.2) 20 (19.6)	0.156
PAM50 --- N (%)				
Luminal A Luminal B HER2 Basal	13 (46.4) 5 (17.9) 8 (28.6) 2 (7.1)	10 (38.5) 5 (19.2) 8 (30.8) 3 (11.5)	19 (37.3) 16 (31.4) 9 (17.6) 7 (13.7)	0.636

Patient were grouped as nulliparous breast cancer (NPBC), pregnancy-associated breast cancer (PABC) and postweaning breast cancer (PWBC). Molecular subtyping was based on prediction analysis of microarray 50 molecular subtype (PAM50)

### Uniform manifold approximation and projection of immune cell profiles

**Fig. 1:**
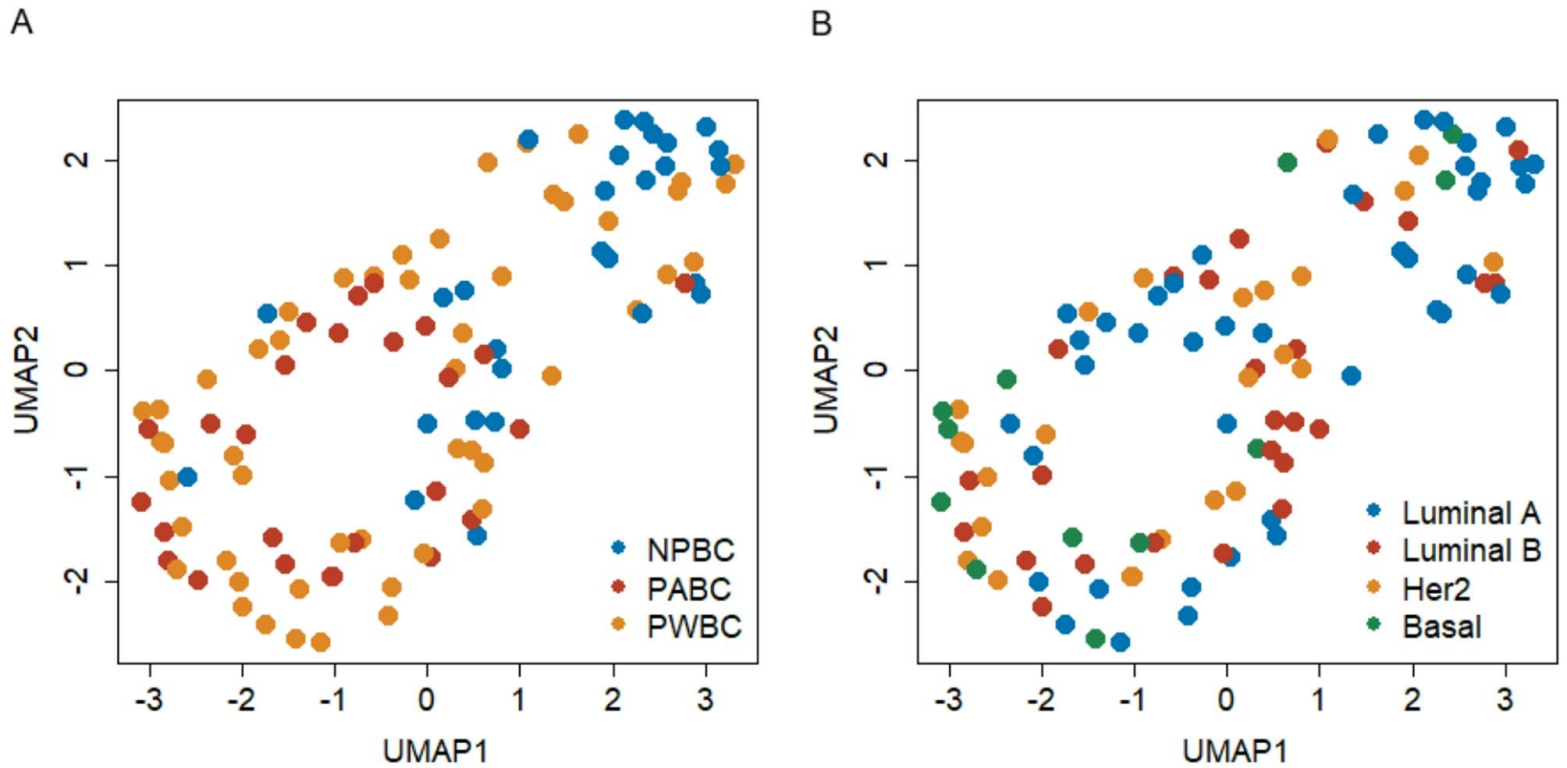
Uniform manifold approximation and projection (UMAP) of immune cell profiles on basis of multiplex IHC and transcriptomic data. Grouped by (**A**) NPBC, PABC, and PWBC, and (**B**) PAM50 subtyping

Uniform manifold approximation and projection (UMAP) was employed to visualize differences in immune cell composition across the study groups based on data derived from both multiplex IHC and transcriptomic analyses. The immune cell distribution patterns in PABC cases closely resembled those observed in the PWBC group (*p* = 0.261, Fig. 1A). However, significant differences were observed between the PABC and NPBC groups (*p* = 0.003) and between the PWBC and NPBC groups (*p* = 0.006). UMAP analysis stratified by PAM50 molecular subtypes revealed no evident differences in immune cell distribution, except for a significant distinction between the luminal A and basal-like subtypes (*p* = 0.006, Fig. 1B). Further subgroup analysis of immune cell composition within each molecular subtype revealed a significant difference between the PABC and NPBC groups within the luminal A subtype (Supplementary Fig. 1). These findings suggest that pregnancy or lactation may modulate the tumor immune microenvironment, particularly in the luminal A subtype of breast cancer.

### Immune scores of breast tumors by pregnancy subgroups

**Fig. 2:**
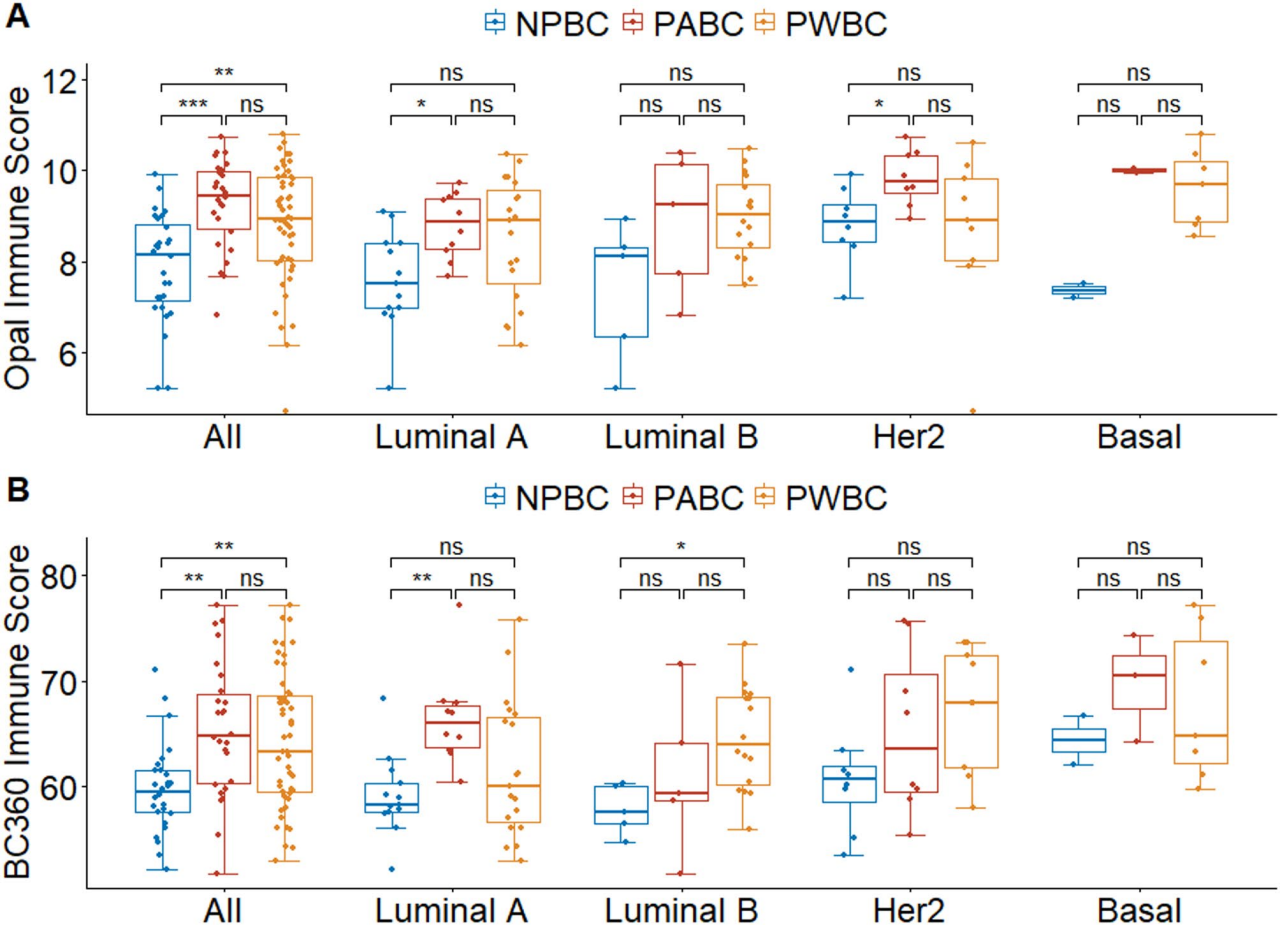
Comparisons of immune scores among subgroups in (**A**) multiplex IHC and (**B**) transcriptome analyses. * *p* < 0.05, ** *p* < 0.01, *** *p* < 0.001, ns non-significant, group difference accessed with Kruskal-Wallis test

Multiplex IHC staining revealed significant differences in Opal immune scores across the NPBC, PABC, and PWBC groups (*p* < 0.001, Fig. 2A). Across all molecular subtypes, tumors in the PABC group had the highest immune scores. In contrast, those in the NPBC group had the lowest. The immune scores were significantly higher in the PABC (*p* < 0.001) and PWBC (*p* = 0.003) groups than in the NPBC group. A subtype-specific analysis further indicated that PABC tumors were associated with significantly higher immune scores than NPBC tumors were in the luminal A (*p* = 0.019) and HER2-enriched (*p* = 0.044, Fig. 2A) subtypes. However, the difference in immune scores between the PWBC and NPBC groups did not reach statistical significance in the subtype-specific analysis, a finding potentially attributed to the limited sample size.

In the transcriptomic analysis, BC360 immune scores significantly differed among the NPBC, PABC, and PWBC groups (*p* < 0.001, Fig. 2B). Across all molecular subtypes, tumors in the PABC group exhibited the highest immune scores. In contrast, those in the NPBC group had the lowest. The BC360 immune scores were significantly higher in the PABC (*p* = 0.011) and PWBC (*p* = 0.002) groups than in the NPBC group. The subtype-specific analysis further revealed that PABC tumors were associated with significantly higher BC360 immune scores than NPBC tumors were in the luminal A subtype (*p* = 0.001). Additionally, PWBC tumors were associated with significantly higher BC360 immune scores than NPBC tumors were in the luminal B subtype (*p* = 0.033).

### Immune cell composition of breast tumors by pregnancy subgroups

**Fig. 3:**
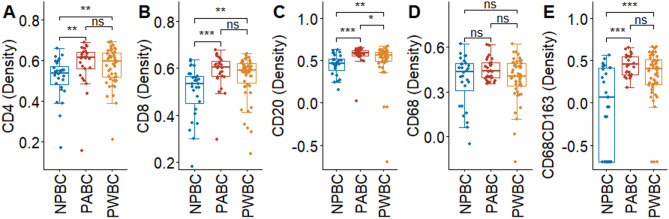
Multiplex IHC immune cell of densities of (**A**) CD4 + T cells, (**B**) CD8 + T cells, (**C**) CD20 + B cells, (**D**) CD68 + cell and (**E**) CD68+/CD163 + M2 cells. * *p* < 0.05, ** *p* < 0.01, *** *p* < 0.001, ns non-significant, group difference accessed with Kruskal-Wallis test

Multiplex IHC analysis revealed that compared with the NPBC subgroup, the PABC subgroup exhibited significantly higher densities of CD4^+^ T cells, CD8^+^ T cells, CD20^+^ B cells, and CD68^+^/CD163^+^ M2 cells (Fig. 3A–C, E). The CD20^+^ B cells density was greater in PABC than PWBC. By contrast, the density of CD68^+^ cells did not significantly differ among the three subgroups (Fig. 3D). The subtype-specific analysis further revealed that compared with NPBC tumors, PABC tumors exhibited increased densities of CD4⁺, CD8⁺, CD20⁺, and CD68⁺/CD163⁺ macrophages in the luminal A subtype; increased densities of CD68⁺/CD163⁺ macrophages in the luminal B subtype; and increased densities of CD8⁺ T cells and CD20⁺ B cells in the HER2-enriched subtype (Supplementary Fig. 2). Additionally, tumors from the PWBC subgroup exhibited higher densities of CD20⁺ B cells and CD68⁺/CD163⁺ macrophages in patients with the luminal A subtype and elevated CD4⁺ T cell densities in patients with the luminal B subtype compared with NPBC tumors (Supplementary Fig. 2). In the luminal B subtype, the density of CD68⁺/CD163⁺ M2 macrophages was higher in PABC compared to PWBC. The spatial colocalization of immune cells and tumor cells is presented in Supplementary Fig. 3. No significant differences in NSInC indices were observed among the NPBC, PABC, and PWBC groups, suggesting that immune–tumor cell colocalization is not influenced by pregnancy status.

**Fig. 4:**
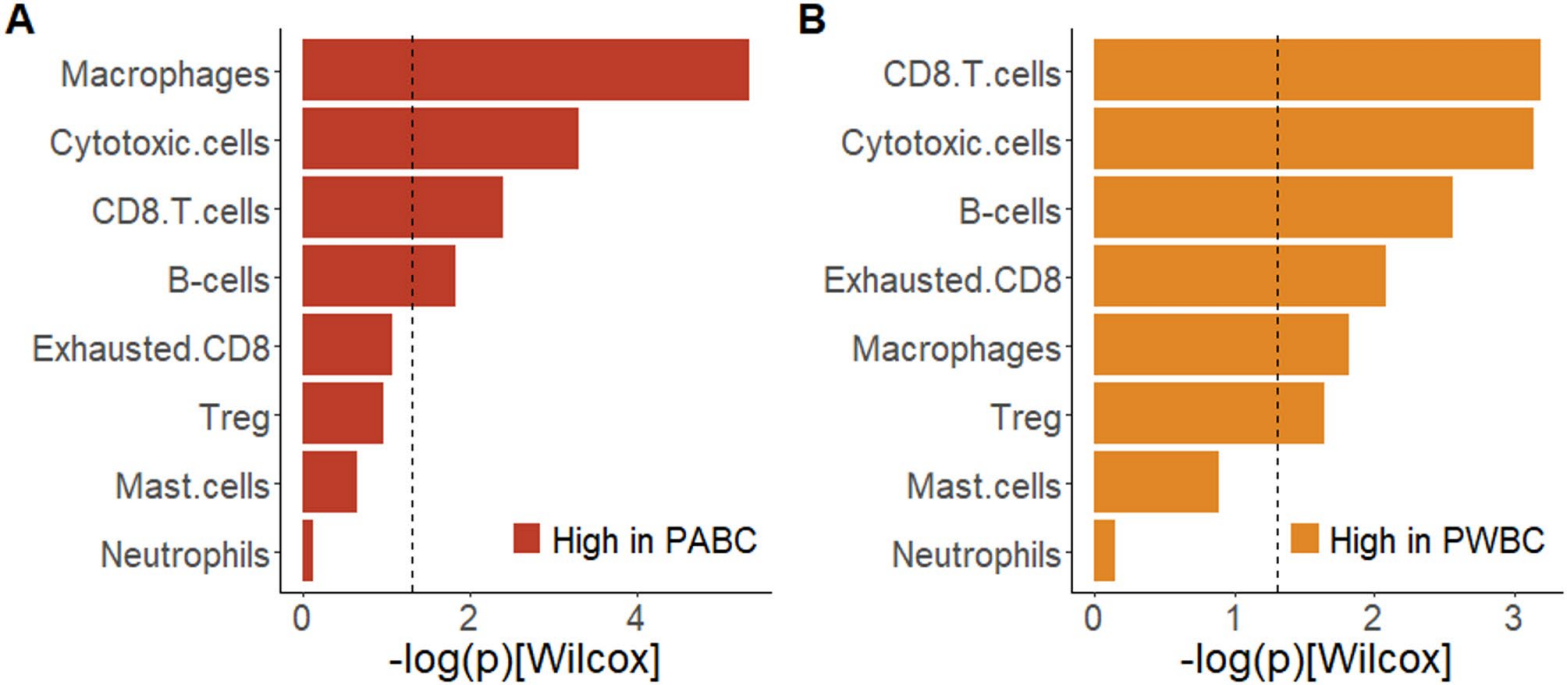
Transcriptomic immune cell signature difference between (**A**) PABC and NPBC, and between (**B**) PWBC and NPBC subgroups. Dotted line indicates *p* value = 0.05

Immune cell signatures derived from the BC360 transcriptomic analysis revealed that the PABC subgroup exhibited higher signatures for macrophages, cytotoxic cells, CD8 T cells, and B cells compared with the NPBC subgroup (Fig. 4A, Supplementary Table 1). Additionally, the PWBC subgroup exhibited higher signatures for cytotoxic cells, CD8 T cells, B cells, exhausted CD8 T cells, macrophages, and regulatory T cells relative to the NPBC subgroup (Fig. 4B, Supplementary Table 1). The macrophage signature was statistically higher in PABC than PWBC (Supplementary Table 1). A transcriptomic analysis stratified by molecular subtype further revealed that compared with the NPBC subgroup, the PABC subgroup exhibited higher signatures for B cells, CD8 T cells, cytotoxic cells, macrophages, and mast cells in the luminal A subtype and a higher macrophage signature in the luminal B and HER2-enriched subtypes. Additionally, compared with the NPBC subgroup, the PWBC subgroup exhibited higher signatures for cytotoxic cells, macrophages, exhausted CD8 T cells, CD8 T cells, and B cells in the luminal B subtype as well as for regulatory T cells, macrophages, and B cells in the HER2-enriched subtype (Supplementary Fig. 4, Supplementary Table 2). The macrophage and mast cell signatures were statistically higher in PABC than PWBC in luminal A subtype (Supplementary Table 2).

Collectively, the findings from the multiplex IHC and transcriptomic analyses indicated a significant increase in B cells, CD8 T cells, and macrophages in the PABC subgroup compared with the NPBC subgroup.

### Validation of immune cell composition by pregnancy subgroup using GEO datasets

A comprehensive search of the GEO database for human expression array data was conducted using the keywords “breast cancer” and “pregnancy,” yielding 22 datasets. After a review of the dataset descriptions, the datasets of 10 studies were excluded because of an absence of pregnancy status information, along with other 3 studies on normal breast tissue, 2 involving cell lines, 1 on post-radiational angiosarcoma, and 1 on ulcerative colitis that contained a duplicated dataset. Additionally, a duplicate entry was identified between dataset GDS4766 and series GSE31192. Three datasets—GSE31192, GSE53031, and GSE112825—were selected for validation. Datasets GSE31192 and GSE112825 were analyzed using the Affymetrix Human Genome U133 Plus 2.0 Array, whereas the GSE53031 dataset was analyzed using the Affymetrix Human Genome U219 Array. In the GSE31192 dataset, PABC was defined as breast cancer diagnosed during pregnancy or within 1 year postpartum, whereas nulliparous women or patients with breast cancer diagnosed 5 years postpartum were categorized into the NPBC group. Only tumor tissue data were included in this validation analysis. Breast cancer cases diagnosed during pregnancy were categorized as PABC, and two age-matched controls were selected for each PABC case from the GSE53031 dataset. Dataset GSE112825 provided the time interval between breast cancer diagnosis and pregnancy, with a 5-year threshold applied to designate PABC status. In total, 93 PABC and 143 NPBC cases were included in the validation cohort. Molecular subtyping revealed a slightly higher proportion of basal-like subtype tumors in the PABC group (Supplementary Table 3).

**Fig. 5:**
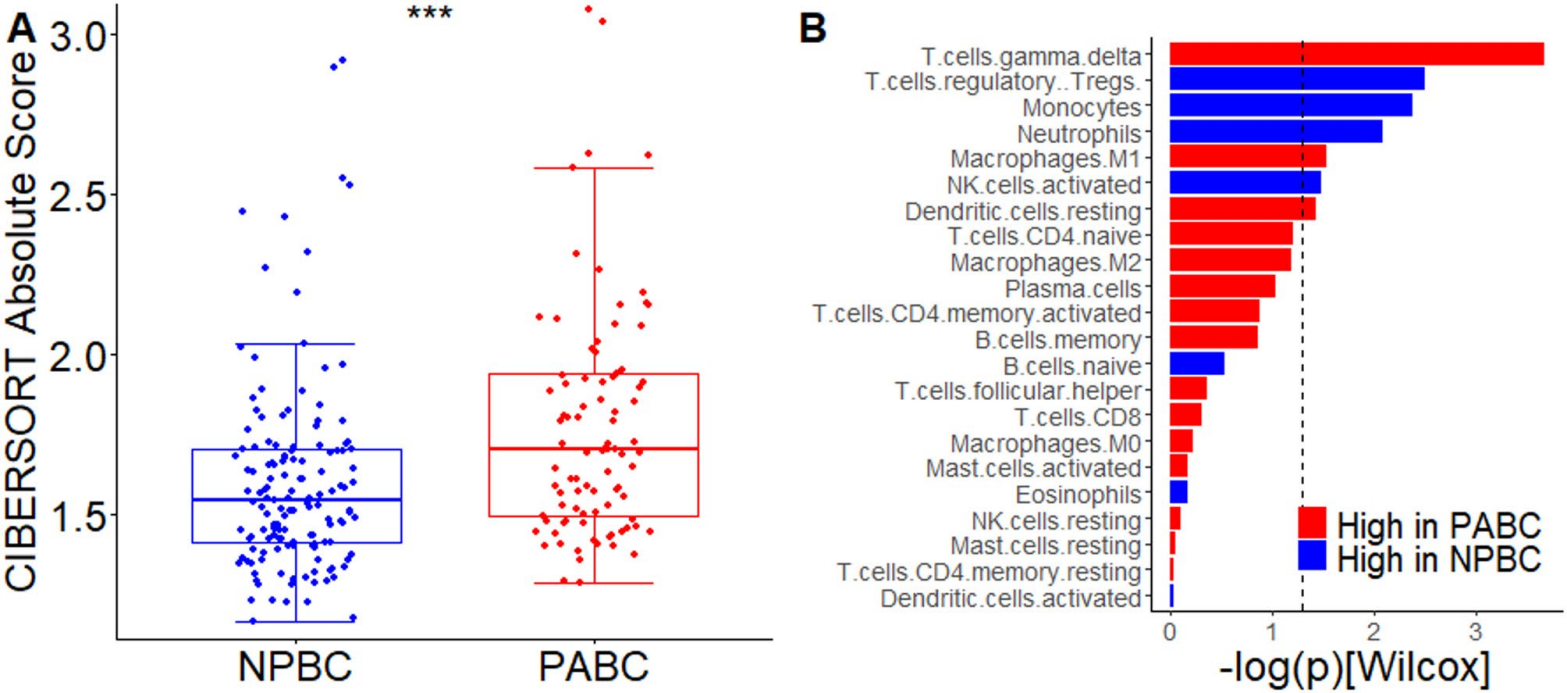
CIBERSORT imputation result of external gene expression datasets in (**A**) immune score and (**B**) immune cell signatures. *** *p* < 0.001, group difference accessed with Wilcoxon rank sum test

CIBERSORT analysis demonstrated significantly higher absolute immune scores in patients with PABC than in those with NPBC (*p* < 0.001, Fig. 5A). The difference could also be found in luminal A, luminal B and basal-like subtypes (Supplementary Fig. 5). Immune cell profiling further revealed that PABC was associated with increased signatures for gamma-delta T cells, M0 macrophages, and resting dendritic cells, whereas signatures for regulatory T cells, monocytes, neutrophils, and activated natural killer cells were decreased (Fig. 5B). The subtype-specific analysis was shown in supplementary Fig. 6. These findings are consistent with results from our study, which indicate that immune cell aggregation occurs during pregnancy but the adaptation of immune cell subsets varies across molecular subtypes.

### Survival impact of tumor immune microenvironment across pregnancy subgroups

The five-year recurrence-free survival (RFS) rate in our cohort was 90.4%. No significant RFS differences were observed among patients with NPBC, PABC, or PWBC (*p* = 0.82; Supplementary Fig. 7A). To assess the prognostic relevance of the immune microenvironment, patients were stratified into high and low immune activity groups based on the median values of immune scores, cell densities, or cell-specific transcriptomic signatures. Log-rank tests showed no significant differences in RFS between patients with high vs. low Opal immune scores (*p* = 0.95) or BC360 immune scores (*p* = 0.50; Supplementary Fig. 7B & C). Univariate Cox regression analyses further confirmed that neither IHC-defined immune cell populations nor transcriptomic immune cell signatures were significantly associated with RFS (Supplementary Table 4).

To investigate the impact of immune cell-tumor colocalization, patients with positive NSInC index were compared to those with zero or negative NSInC index. While not reaching statistical significance, higher colocalization between CD4 + or CD8 + T cells and tumor cells showed trends toward improved RFS (*p* = 0.064 and *p* = 0.071, respectively; Supplementary Table 5).

## Discussion

This study employed a dual-methodological approach to evaluate the tumor immune microenvironment and demonstrated that PABC was characterized by significantly higher immune cell abundance compared with NPBC, with this effect persisting during the weaning period. IHC analysis revealed that PABC was associated with increased densities of CD4^+^, CD8^+^, CD20^+^, and CD68^+^CD163^+^ cells. Additionally, the transcriptomic analysis indicated higher signatures for macrophages, cytotoxic cells, CD8^+^ T cells, and B cells. These findings were validated across three external gene expression datasets. Notably, patients with PWBC generally exhibited immune scores and immune cell compositions that were intermediate between those of the PABC and NPBC groups, particularly within the luminal A subtype. This pattern suggests that the immunological reaction associated with pregnancy may progressively attenuate after delivery.

The observed increase in CD8⁺ T-cell infiltration and elevated cytotoxic T-cell gene signatures in PABC suggest a potential enhancement of anti-tumor immunity. However, the concurrent enrichment of CD68⁺CD163⁺ tumor-associated macrophages and upregulation of macrophage-related signatures indicate a possibly immunosuppressive microenvironment. Moreover, despite differences in immune cell densities, spatial analysis revealed no significant variation in the colocalization of immune and tumor cells between the pregnancy and postpartum periods. This paradoxical immune landscape may reflect the complex and dynamic immune remodeling associated with pregnancy, which could simultaneously promote immune activation and tolerance. These findings highlight the need for functional validation to delineate the net impact of these immune alterations on tumor progression and patient outcomes.

During pregnancy, the immune system undergoes dynamic remodeling to enhance maternal tolerance toward the fetus while preserving its protective functions. The innate immunity system, which operates in a nonspecific manner, rapidly responds to pathogens and facilitates antigen presentation to T cells, thereby initiating the adaptive immune response. Immune responses are classified based on the subtype of helper T cells (Th) involved as Th1- or Th2-mediated [18]. Th1 T cells activate M1 macrophages and CD8^+^ T cells, thereby supporting cellular immunity, which plays a role in autoimmune disease and tumor suppression [19]. Conversely, Th2 responses facilitate humoral immunity by activating B cells, eosinophils, and mast cells, processes associated with allergic reactions and tumor progression [19]. Therefore, despite being a foreign antigen, the fetus is naturally protected from maternal immune rejection.

A previous study proposed that a shift from a Th1- to Th2-dominant immune environment occurs during pregnancy [20]. Additionally, the upregulation of regulatory T cells and programmed death receptor-1 ligand 1 contributes to immune tolerance during pregnancy [21, 22]. These immunological adaptations contribute to the establishment of a tumorigenic microenvironment. A study evaluating hematoxylin and eosin (H&E)-stained pathology slides from 86 PABC cases diagnosed during pregnancy and 116 NPBC cases reported reduced tumor-infiltrating lymphocytes (TILs) in the PABC group [23]. Recent study revealed relative decreased TILs in 15 PABCs during pregnancy and higher TILs in 18 PABCs in the first postpartum year [24]. However, another study reported no significant differences in H&E-stained TILs among NPBC, PABC, and PWBC groups [25]. Furthermore, a separate H&E-based study reported increased TILs in PABC during pregnancy [26]. Although the International TILs Working Group advocates for H&E-based TIL analyses, the method is associated with substantial interobserver variability, which compromises its accuracy [27]. A transcriptome-based analysis reported a high adaptive immune score in PWBC cases diagnosed within 10 years postpartum [28]. In the present study, both multiplex IHC and transcriptomic analyses revealed higher immune scores in the PABC and PWBC groups, underscoring the reliability and reproducibility of automated methodologies.

An IHC-based study reported the presence of CD4^+^, CD8^+^, and FOXP3^+^ T cells in 31.9%, 81.9%, and 34.5% of PABC cases during pregnancy, respectively, compared with in 48.3%, 68.5%, and 23.6% of NPBC cases, respectively [26]. Another transcriptome-based study showed relative increased B cell, macrophage, neutrophil and regulatory T cell signature in PABC diagnosed within 1 year postpartum compared to those diagnosed during pregnancy [24]. In the present study, PABC was associated with increased infiltration of B cells, CD8^+^ T cells, and macrophages. Typically, increased infiltration of CD8^+^ T cells is indicative of a Th1-dominant immune environment, whereas increased levels of regulatory T cells and B cells are characteristic of a Th2-skewed immune environment. This duality complicates the classification of its immune environment in PABC. A transcriptome-based study reported increased signatures for T cells, B cells, CD8^+^ T cells, and follicular helper cells in PWBC cases diagnosed within 10 years postpartum compared with nulliparous controls [28]. Another study using multiplex IHC and RNA sequencing revealed higher densities and signatures for B cells, CD8 T cells, and plasma cells in the PWBC group than in the NPBC group [25].

In the present study, PWBC cases exhibited increased infiltration of B cells, CD4^+^ T cells, CD8^+^ T cells, and macrophages, displaying an intermediate immune profile between those of NPBC and PABC. This pattern contrasts with previously reported findings [25]. Lefrere et al. observed no significant differences in survival outcomes between NPBC and PWBC after stratification by levels of CD8, CD20, and immunoglobulin expression. However, among patients with high CD38 expression, those in the PWBC group had a poorer prognosis in their report [25]. Additionally, PWBC cases exhibited increased IgA expression and reduced IgG expression, suggesting that altered immunoglobulin expression may contribute to their adverse clinical outcomes. In a previous IHC-based study, high CD4⁺ and CD8⁺ TILs were associated with improved disease-free survival in NPBC patients, but not in those with PABC [26]. In contrast, our study found no significant association between recurrence-free survival and immune scores, IHC-derived cell densities, or transcriptomic immune cell signatures. However, spatial analysis revealed a trend toward better outcomes associated with higher colocalization indices of CD4⁺ and CD8⁺ T cells with tumor cells.

This study has several limitations that should be acknowledged. First, the sample size was relatively small for the luminal B, HER2-enriched, and basal-like subtypes, resulting in reduced representativeness and low statistical power in these groups. The limited sample size constrained the statistical power needed for multivariate survival analysis; therefore, only univariate analyses were conducted. As such, the findings should be interpreted as exploratory in nature. To mitigate this limitation, three GEO datasets were incorporated into the validation cohort. The validation results exhibited only moderate consistency with those of our cohort, which may be attributable to methodological differences. Second, other variables, such as patient age, breastfeeding status, and parity, were not accounted for in the analysis. Breastfeeding directly modifies breast tissue, and multiparity has been associated with enhanced immune responses [29], both of which may contribute to the immunological characteristics of PABC. Third, heterogeneity in immune cell definitions complicates cross-study comparisons. Although comparable results were observed between the two methods, it should be interpreted carefully. For example, CD68^+^ monocyte lineages and CD68^+^CD163^+^ M2 macrophages were assessed using a colorimetric IHC kit, whereas the RNA-based macrophage signature included CD68, CD84, and CD163 expression. Hence, the CD68^+^ cell density from IHC was not entirely equivalent to the macrophage signature from BC360 panel and so did other immune cell subsets. To account for these differences, analyses from Opal Polaris and BC360 were treated as distinct entities and discussed separately.

## Conclusion

This study revealed increased immune cell infiltration in PABC. Pregnancy alters the immune microenvironment of breast cancer by increasing the number of both tumor-suppressive cytotoxic cells and tumor-promoting macrophages and plasma cells. To further elucidate the complex immunological landscape associated with pregnancy, future large-scale studies should incorporate spatial analysis of immune and tumor cells, immune-modulatory factors such as cytokines, specific immune cell subpopulations, and immune receptor status.

## Supplementary Information

Below is the link to the electronic supplementary material.

Supplementary Material 1

## Data Availability

The datasets used and/or analyzed during the current study are available from the corresponding author on reasonable request.
