# Supplementary material for: Characterization of the tumor immune microenvironment in pregnancy-associated breast cancer through multiplex immunohistochemistry and transcriptome analyses
Source: Breast Cancer Res. 2025 Aug 26;27:154. doi: 10.1186/s13058-025-02097-4 (PMC12382146; doi:10.1186/s13058-025-02097-4)
Supplement: Supplementary file 1 — Supplementary Material 1 [file 13058_2025_2097_MOESM1_ESM.docx]

Supplementary Figure 1 Molecular subtype-specific UMAP immune cells analysis


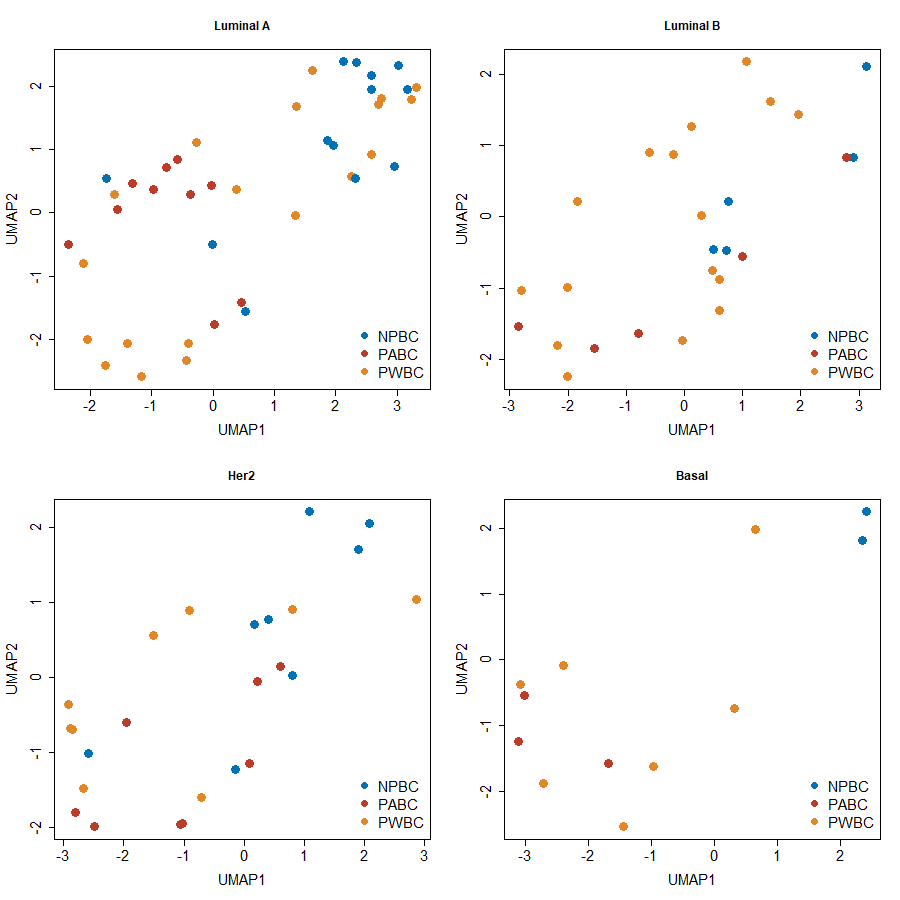
 Supplementary Figure 2 Comparisons of immune cell compositions among NPBC, PABC, and PWBC subgroups in multiplex IHC analysis across each molecular subtype


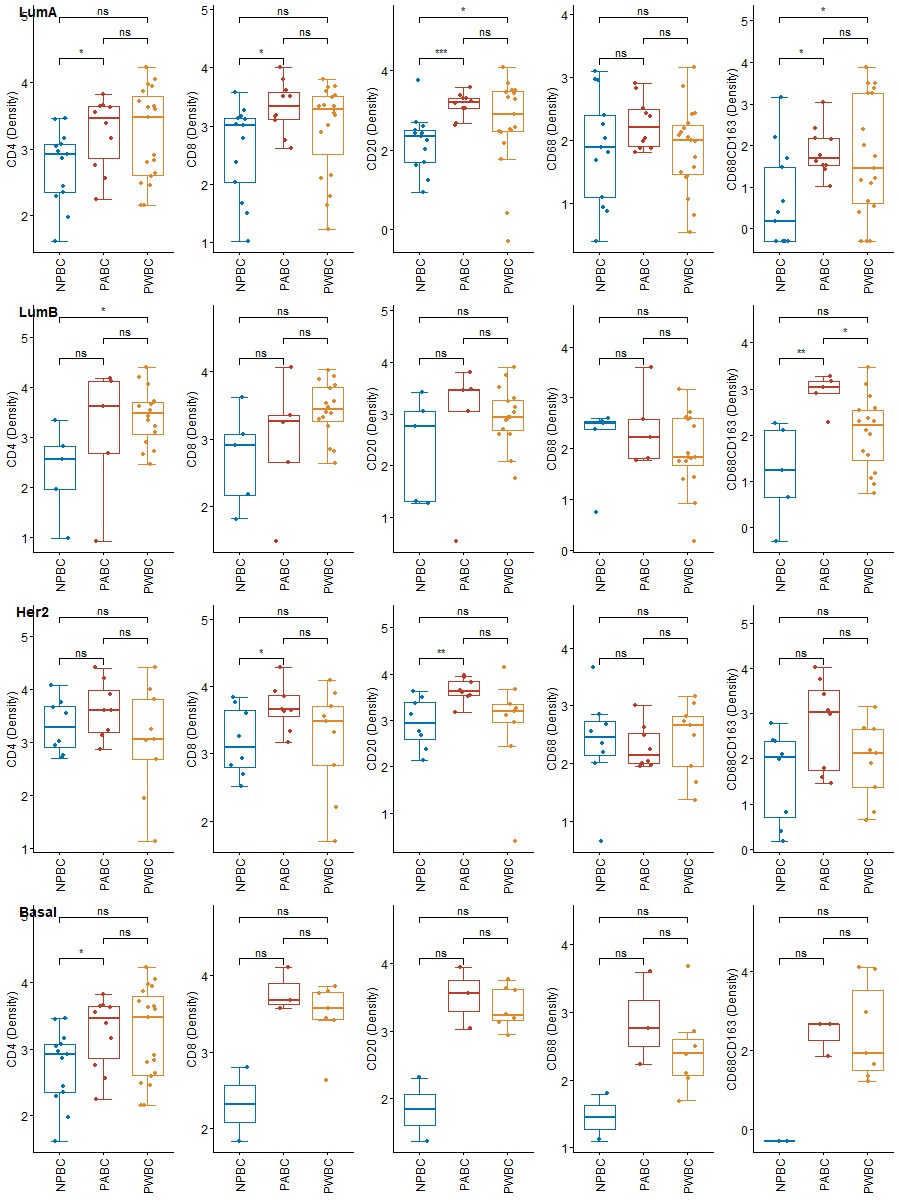


Supplementary Figure 3 Colocalization of immune cells and tumors among NPBC, PABC, and PWBC


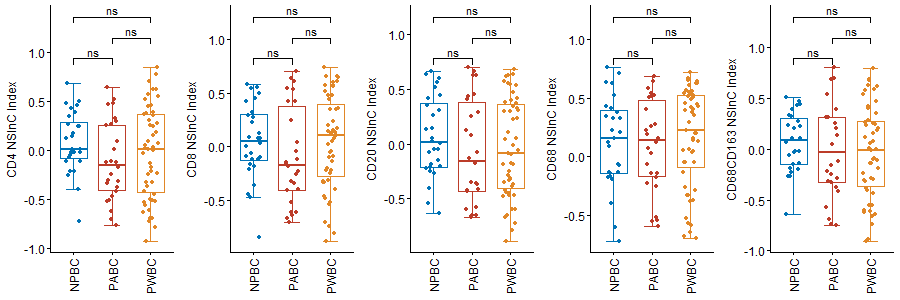


Supplementary Figure 4 Transcriptomic analysis of immune cells across each molecular subtype


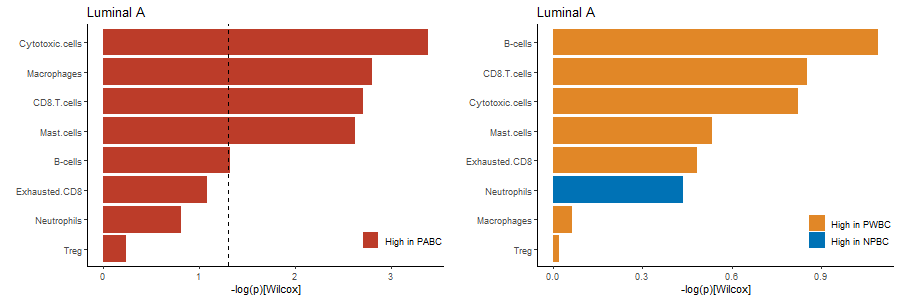


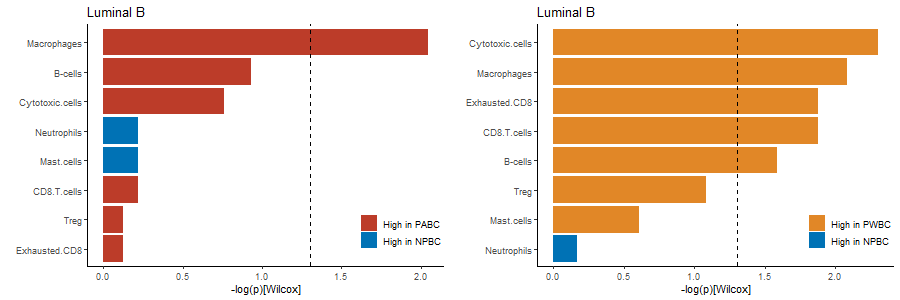

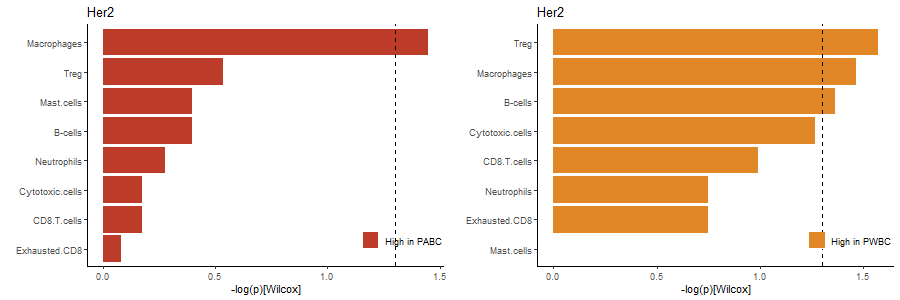

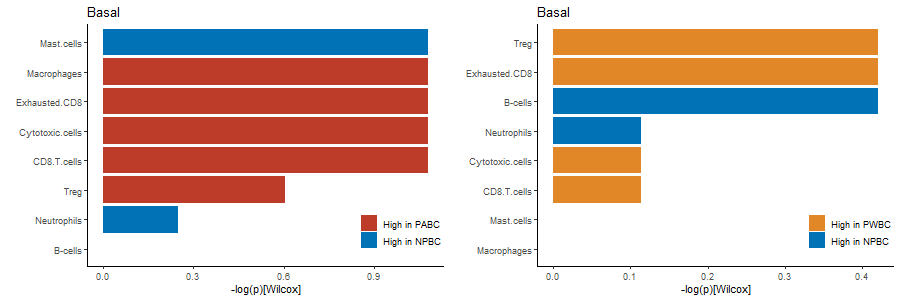


Supplementary Figure 5 CIBERSORT immune score in external datasets


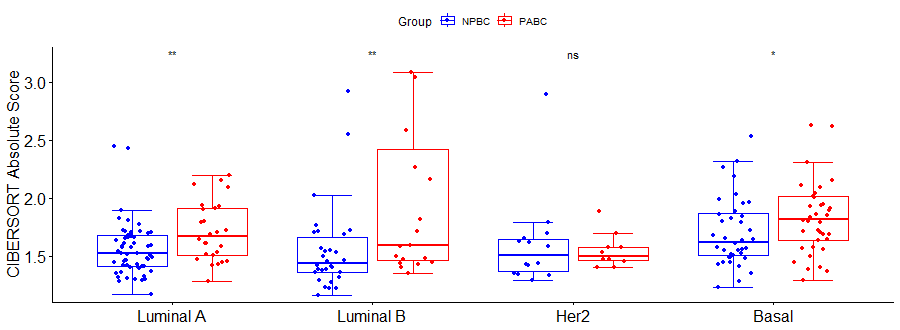


Supplementary Figure 6 CIBERSORT immune cells in external datasets


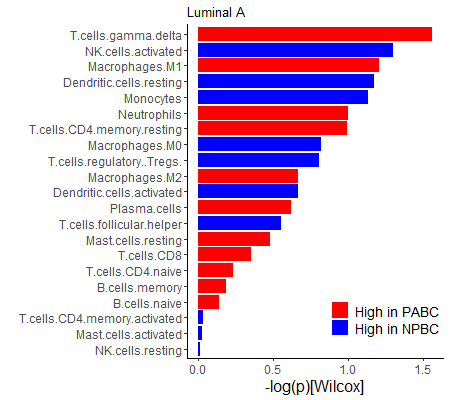

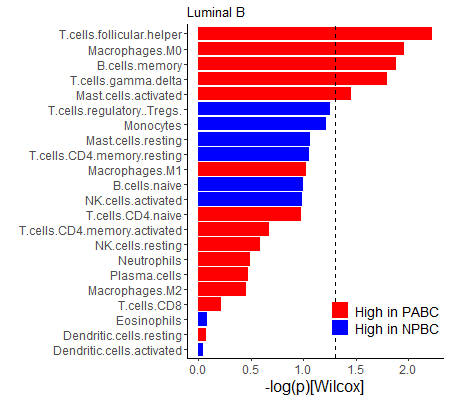

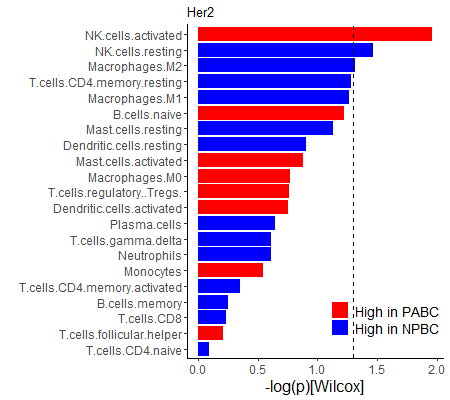

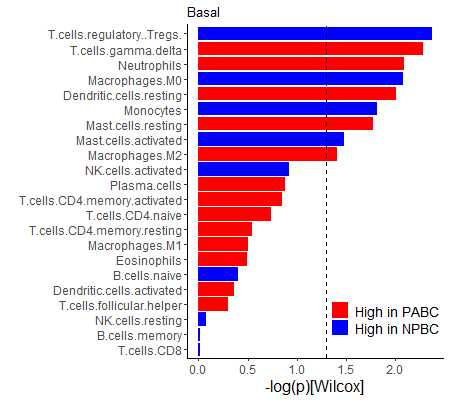


Supplementary Figure 7 Comparisons of recurrence-free survival among NPBC, PABC, and PWBC (A), between low and high Opal immune score (B), and between low and high BC360 immune score (C)

| **(A)** | **(B)** | **(C)** |
| --- | --- | --- |
| 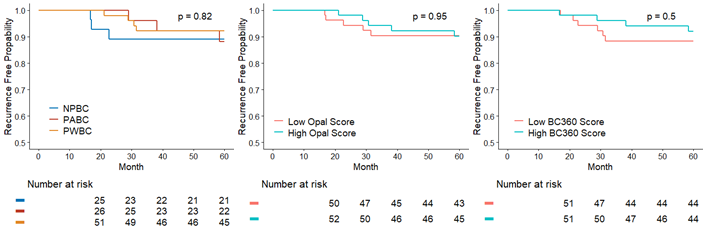 | | |

Supplementary Table 1 Comparisons of BC360 immune cell signatures among NPBC, PABC, and PWBC

| All case | 1.NPBC  Mean (SD) | 2.PABC  Mean (SD) | 3.PWBC  Mean (SD) | Kruskal  Wallis *p* | Post hoc |
| --- | --- | --- | --- | --- | --- |
| Cytotoxic cells | 6.5 (0.7) | 7.6 (1.2) | 7.4 (1.2) | <0.001 | 2 > 1; 3 > 1 |
| Treg | 6.7 (0.6) | 7.1 (1.1) | 7.2 (1.1) | 0.040 | 3 > 1 |
| Macrophages | 9.6 (0.6) | 10.5 (0.5) | 10.0 (0.8) | <0.001 | 2 > 1; 2 > 3; 3 > 1 |
| Exhausted CD8 | 7.1 (0.7) | 7.9 (1.4) | 7.7 (1.3) | 0.034 | 3 > 1 |
| Mast cells | 7.1 (0.9) | 7.6 (1.3) | 7.5 (1.2) | 0.281 |  |
| CD8 T cells | 7.2 (0.7) | 8.1 (1.3) | 8.1 (1.2) | 0.001 | 2 > 1; 3 > 1 |
| B-cells | 6.1 (1.1) | 6.9 (1.2) | 7.0 (1.4) | 0.007 | 2 > 1; 3 > 1 |
| Neutrophils | 9.6 (0.8) | 9.8 (1.0) | 9.6 (0.9) | 0.895 |  |

Supplementary Table 2 Comparisons of BC360 immune cell signatures among NPBC, PABC, and PWBC, subgroup, stratified by molecular subtype

|  | 1.NPBC  Mean (SD) | 2.PABC  Mean (SD) | 3.PWBC  Mean (SD) | Kruskal  Wallis *p* | Post hoc |
| --- | --- | --- | --- | --- | --- |
| **Luminal A** |  |  |  |  |  |
| Cytotoxic cells | 6.3 (0.6) | 7.6 (0.8) | 6.8 (1.1) | 0.006 | 2 > 1 |
| Treg | 6.5 (0.5) | 6.7 (0.9) | 6.5 (1.0) | 0.906 |  |
| Macrophages | 9.5 (0.6) | 10.7 (0.7) | 9.6 (0.7) | 0.001 | 2 > 1; 2 > 3 |
| Exhausted CD8 | 6.8 (0.7) | 7.5 (1.0) | 7.1 (0.8) | 0.209 |  |
| Mast cells | 7.3 (0.7) | 8.6 (0.8) | 7.8 (1.1) | 0.010 | 2 > 1; 2 > 3 |
| CD8 T cells | 7.1 (0.7) | 8.2 (0.9) | 7.6 (1.2) | 0.018 | 2 > 1 |
| B-cells | 6.0 (1.0) | 6.9 (0.9) | 6.6 (1.3) | 0.049 | 2 > 1 |
| Neutrophils | 9.7 (0.6) | 10.3 (1.0) | 9.6 (0.9) | 0.150 |  |
| **Luminal B** |  |  |  |  |  |
| Cytotoxic cells | 6.1 (0.4) | 7.1 (1.4) | 7.4 (0.9) | 0.022 | 3 > 1 |
| Treg | 6.4 (0.5) | 6.7 (1.6) | 7.3 (1.0) | 0.236 |  |
| Macrophages | 9.3 (0.2) | 10.1 (0.4) | 10.1 (0.6) | 0.015 | 2 > 1; 3 > 1 |
| Exhausted CD8 | 6.9 (0.4) | 7.4 (1.8) | 7.7 (1.1) | 0.045 | 3 > 1 |
| Mast cells | 7.2 (0.6) | 6.9 (1.0) | 7.8 (0.8) | 0.181 |  |
| CD8 T cells | 6.9 (0.3) | 7.4 (1.7) | 8.1 (0.9) | 0.031 | 3 > 1 |
| B-cells | 5.4 (0.3) | 6.3 (1.4) | 6.6 (1.3) | 0.045 | 3 > 1 |
| Neutrophils | 9.7 (1.4) | 9.3 (0.5) | 9.5 (0.6) | 0.686 |  |
| **Her2-enriched** |  |  |  |  |  |
| Cytotoxic cells | 6.9 (0.8) | 7.5 (1.5) | 8.0 (1.1) | 0.241 |  |
| Treg | 7.0 (0.5) | 7.5 (0.9) | 7.7 (0.5) | 0.049 | 3 > 1 |
| Macrophages | 9.8 (0.7) | 10.4 (0.4) | 10.4 (0.4) | 0.029 | 2 > 1; 3 > 1 |
| Exhausted CD8 | 7.5 (0.7) | 7.9 (1.3) | 8.4 (1.3) | 0.409 |  |
| Mast cells | 6.7 (1.5) | 7.5 (1.1) | 6.8 (1.6) | 0.435 |  |
| CD8 T cells | 7.4 (1.0) | 7.8 (1.3) | 8.5 (1.2) | 0.254 |  |
| B-cells | 6.2 (1.2) | 6.8 (1.5) | 7.9 (1.8) | 0.042 | 3 > 1 |
| Neutrophils | 9.2 (0.9) | 9.7 (1.1) | 9.9 (1.0) | 0.382 |  |
| **Basal-like** |  |  |  |  |  |
| Cytotoxic cells | 7.4 (0.1) | 8.9 (1.0) | 8.0 (1.6) | 0.413 |  |
| Treg | 7.2 (0.6) | 8.0 (1.0) | 7.9 (1.1) | 0.551 |  |
| Macrophages | 10.1 (0.1) | 10.8 (0.4) | 10.4 (1.1) | 0.551 |  |
| Exhausted CD8 | 7.6 (0.1) | 9.9 (1.1) | 8.9 (1.9) | 0.291 |  |
| Mast cells | 6.8 (0.8) | 5.5 (0.2) | 6.9 (1.0) | 0.104 |  |
| CD8 T cells | 8.0 (0.1) | 9.4 (1.4) | 8.8 (1.4) | 0.413 |  |
| B-cells | 7.8 (1.0) | 8.1 (0.5) | 7.5 (0.9) | 0.476 |  |
| Neutrophils | 9.4 (0.4) | 9.1 (1.1) | 9.4 (1.0) | 0.898 |  |

Supplementary Table 3 Distribution of molecular subtyping in external datasets

|  | NPBC  n=145 | PABC  n=93 | *p* value |
| --- | --- | --- | --- |
| PAM50 (%)  Luminal A  Luminal B  HER2  Basal | 61 (42.1)  30 (20.7)  14 (9.6)  40 (27.6) | 26 (27.9)  19 (20.4)  10 (10.8)  38 (40.9) | 0.101 |

Supplementary Table 4 Impact of immune cells on recurrence free survival (High vs Low)

| Opal IHC cell | HR | 95%C.I. | P | BC360 signature | HR | 95%C.I. | P |
| --- | --- | --- | --- | --- | --- | --- | --- |
| CD4 | 0.65 | 0.19 – 2.28 | 0.506 | B cell | 1.01 | 0.29 – 3.45 | 0.985 |
| CD8 | 0.62 | 0.18 – 2.13 | 0.449 | CD8 T cell | 0.41 | 0.11 – 1.56 | 0.192 |
| CD20 | 0.96 | 0.28 – 3.27 | 0.952 | Cytotoxic cell | 0.41 | 0.11 – 1.56 | 0.192 |
| CD68 | 0.97 | 0.59 – 3.32 | 0.967 | Exhausted CD8 | 0.41 | 0.11 – 1.54 | 0.185 |
| CD68CD163 | 1.51 | 0.43 – 5.27 | 0.521 | Macrophage | 1.03 | 0.30 – 3.55 | 0.958 |
|  |  |  |  | Mast cell | 1.53 | 0.44 – 5.37 | 0.506 |
|  |  |  |  | Neutrophil | 0.67 | 0.19 – 2.34 | 0.529 |
|  |  |  |  | Treg | 0.96 | 0.28 – 3.27 | 0.952 |

Supplementary Table 5 Impact of colocalization between immune and tumor cells on recurrence free survival (NSInC index > 0 vs NSInC index ≤ 0)

| Immune cell | HR | 95%C.I. | P |
| --- | --- | --- | --- |
| CD4 | 0.24 | 0.05 – 1.09 | 0.064 |
| CD8 | 0.24 | 0.05 – 1.12 | 0.071 |
| CD20 | 0.49 | 0.13 – 1.88 | 0.299 |
| CD68 | 2.32 | 0.50 – 10.7 | 0.281 |
| CD68CD163 | 1.03 | 0.30 – 3.48 | 0.966 |
